# Reliability and Validity of the Japanese Version of the Internet Gaming Disorder Scale for Children (IGDS‐C)

**DOI:** 10.1002/npr2.12518

**Published:** 2025-01-10

**Authors:** Koki Ono, Makoto Tokushige, Nanami Hiratani, Kyosuke Kaneko, Toshitaka Hamamura, Yuki Miyamoto, Masaru Tateno, Masaya Ito, Ayumi Takano

**Affiliations:** ^1^ National Center of Neurology and Psychiatry National Institute of Mental Health Kodaira Tokyo Japan; ^2^ Department of Psychiatric Nursing, Graduate School of Medicine The University of Tokyo Tokyo Japan; ^3^ Department of Mental Health and Psychiatric Nursing Institute of Science Tokyo Tokyo Japan; ^4^ National Center of Neurology and Psychiatry National Center for Cognitive Behavior Therapy and Research Kodaira Tokyo Japan; ^5^ Tokiwa Child Development Center Tokiwa Hospital Sapporo Japan; ^6^ Department of Neuropsychiatry, School of Medicine Sapporo Medical University Sapporo Japan

**Keywords:** adolescents, children, Internet Gaming Disorder, reliability, validity

## Abstract

**Aim:**

The Internet Gaming Disorder Scale is a 9‐item screening instrument developed based on the diagnostic criteria for Internet Gaming Disorder (IGD) in the DSM‐5. This study aimed to examine the reliability and validity of the Internet Gaming Disorder Scale for children (IGDS‐C) in Japanese clinical and nonclinical populations.

**Methods:**

The study included clinical outpatients aged 9–29 with problematic game use and nonclinical adolescents aged 12–18 who played online games at least once a week. Reliability was examined by calculating internal consistency and test–retest reliability. Validity was assessed using Spearman's correlation and Confirmatory Factor Analysis (CFA).

**Results:**

A total of 746 participants (93 clinical, 653 nonclinical) were eligible for statistical analysis. Reliability results revealed acceptable internal consistency (Cronbach's *α* = 0.87) and test–retest reliability (intraclass correlation coefficient = 0.62). CFA results (Comparative Fit Index = 0.92, Tucker–Lewis Index = 0.90, root mean square error of approximation = 0.10, standardized root mean square residual = 0.05, factor loadings = 0.59–0.71) and significant correlations with the GAMES test, psychological distress, and gaming hours verified the validity of the IGDS‐C.

**Conclusion:**

The study verified the reliability and validity of the IGDS‐C in Japanese clinical and nonclinical participants, suggesting that it generally reflects the severity of IGD well.

## Abstract 2

This study assesses the reliability and validity of the Japanese version of the Internet Gaming Disorder Scale for children (IGDS‐C) and provides evidence supporting the use of IGDS‐C among Japanese clinical and nonclinical populations.

## Graphical abstract

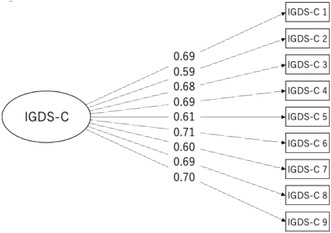

## 1 Introduction

Internet Gaming Disorder (IGD) is a significant global issue characterized by excessive and compulsive use of internet games [1, 2]. Studies have shown that the prevalence of IGD among adolescents and young adults is increasing, with reported rates varying from 4.1% to 24.0% across different regions and cultures [3]. Evidence from various studies underscore the urgent necessity to address this disorder, as its impact on youth can result in detrimental effects on mental health, educational performance, and social interactions [4, 5].

The Internet Gaming Disorder Scale (IGDS) is a 9‐item, dichotomous screening instrument based on the diagnostic criteria for Internet gaming disorder in the DSM‐5 [1, 6]. Previous studies have verified its ability to distinguish between normal, risky, and disordered gamers, providing a nuanced understanding of gaming behaviors [6]. Among the various scales developed for IGD, the IGDS is characterized by the development of a corresponding Parental version of the Internet Gaming Disorder Scale (PIGDS) as an external rating method [7]. This is crucial in treatments, where the patients are young, and often the first point of contact is likely a parent or close relative [7]. The IGDS has also been rigorously tested on a representative sample of adolescents and adults, demonstrating robust psychometric properties including internal consistency and criterion‐related validity [6, 8]. Multiple language versions have been developed and confirmed to have good reliability and validity among internet game users as well [9, 10, 11]. The IGDS is a promising screening instrument that can be used for both research and diagnostic purposes.

An Internet Gaming Disorder Scale Japanese Version (IGDS‐J) was developed by Sumi in 2018, with a preliminary investigation on reliability in a Japanese population [12]. However, the target population consisted of middle school and university students, excluding elementary school children [12]. Japanese “kanji” characters vary greatly in difficulty with the majority of characters not yet taught in elementary school, making the wording of the questions in the IGDS‐J potentially unsuitable for use by children. Thus, the Japanese version of the Internet Gaming Disorder Scale for children (IGDS‐C) was developed with permission from the author of the IGDS‐J to accommodate elementary school children as well [13]. Although the scale was evaluated for linguistic validity among elementary school outpatients aged 9–12 with problematic game use [13], overall reliability and validity are yet to be confirmed. The purpose of this study was to widely examine its reliability and validity across clinical and nonclinical populations in Japan.

## 2 Methods

### 2.1 Participants and Procedure

This study was a cross‐sectional survey targeting clinical and nonclinical participants. Clinical participants were recruited in October 2021 through their physicians in medical institutions that provide treatment for Gaming Disorder or are members of The Japanese Society for Child and Adolescent Psychiatry. The inclusion criteria were outpatients (ages 9–29) with problematic gaming. Nonclinical participants were recruited in June 2023 through an online research company and included adolescents aged 12–18 who played online games at least once a week. Surveys for clinical participants were administered as paper surveys through their physicians, while nonclinical participants completed an online survey. A test–retest survey was conducted for clinical participants who responded to the first survey within 4 weeks. Test–retest survey for nonclinical participants was administered to all participants 1 week after the first survey.

### 2.2 Measures

#### 2.2.1 Sociodemographic Characteristics

Information regarding sex, age, and gaming hours (weekdays and holidays) were collected as demographic data and participant characteristics.

#### 2.2.2 Internet Gaming Disorder Scale for Children (IGDS‐C)

The IGDS‐C consists of nine items and a dichotomous (yes/no) response format [13]. Scores range from 0 to 9, with higher scores indicating greater severity of IGD. The original version of the IGDS determined the cutoff to be five points [6].

#### 2.2.3 GAMing Engagement Screening Test (GAMES Test)

The GAMES test was developed based on the ICD‐11 diagnostic criteria for Gaming Disorder [14]. It consists of eight dichotomous (yes/no) questions and one trichotomous (~2 h/2–6 h/~6 h) question on gaming duration. Higher scores indicate greater severity of IGD, with a maximum score of 10 points. The GAMES test has been verified for reliability and validity in a Japanese general population, with a cutoff determined to be five points [14].

#### 2.2.4 Psychological Distress

Clinical participants aged 9–12 were evaluated using the Birleson Depression Self‐Rating Scale for Children (DSRS‐C) with a maximum score of 18 [15]. The General Health Questionnaire 30‐item (GHQ30) which has a maximum score of 30 was utilized for clinical participants older than 13 years [16]. Nonclinical participants were assessed using The Kessler Psychological Distress Scale 6‐item (K6), with a maximum score of 24 [17]. All scales indicated a higher degree of psychological distress with higher scores.

### 2.3 Statistical Analysis

First, a Directed Questions Scale (DQS) was used to exclude responses from nonclinical participants who did not provide satisfactory answers due to factors such as carelessness and satisficing [18]. Next, analysis of the IGDS‐C was conducted for clinical and nonclinical participants separately as well as for the combined group.

Reliability of the IGDS‐C was assessed by calculating the internal consistency (Cronbach's *α* coefficient) and test–retest reliability (Intraclass Correlation Coefficient, ICC). Cronbach's *α* is calculated by dividing the sum of each item score variance by the total score variance, adjusting for item numbers [19]. ICC is calculated by dividing the mean square variance between subjects by the total variance [20]. Confirmatory factor analysis (CFA) was conducted as a statistical technique that tests whether the observed items align with a hypothesized factor structure, assessed utilizing goodness of fit indices (GFI). Cutoff standards for GFI were Comparative Fit Index (CFI) ≥ 0.95, Tucker–Lewis Index (TLI) > 0.95, root mean square error of approximation (RMSEA) < 0.06, and standardized root mean square residual (SRMR) ≤ 0.08 [21]. Factor loadings, which are calculated by optimizing the correlations between observed items and their latent factors, were reported to represent the relationship strength of each item. Concurrent and convergent validity was examined through Spearman's correlation between the total scores of the IGDS‐C with the GAMES test, scales relating to psychological distress, and gaming hours (weekdays and holidays). As there were possible differences in demographic characteristics of the clinical and nonclinical participants, *t*‐tests were performed to confirm differences between participant groups. All data analyses were conducted using R version 4.3.1 (packages: ltm, psych, lavaan).

## 3 Results

A total of 746 participants (93 clinical, 653 nonclinical) completed the survey and were eligible for statistical analysis. Table 1 presents the demographic information and results of the gaming screening tests among the participants. Clinical participants were significantly more male and older compared to the nonclinical sample. IGDS‐C responses also revealed that clinical participants scored significantly higher than nonparticipants, except for Q7 (Have you hidden the time you spend on games from others?). GAMES test total score, weekday gaming hours, and holiday gaming hours were also significantly higher in the clinical group.

**TABLE 1:** Demographic information and clinical screening results of the participants (*N* = 746).

	All (*n* = 746)	Clinical (*n* = 93)	Nonclinical (*n* = 653)	
	*n* (%)/mean (SD)	*n* (%)/mean (SD)	*n* (%)/mean (SD)	*p*
Sex (male)	481 (64.5%)	73 (78.5%)	408 (62.5%)	< 0.01
Age (years)	14.5 (± 2.7)	17.0 (± 5.4)	14.2 (± 1.8)	< 0.01
IGDS‐C total score	2.9 (± 2.9)	4.6 (± 2.7)	2.7 (± 2.9)	< 0.01
Q1. Have there been periods when all you could think of was the moment that you could play a game? (yes)	249 (33%)	60 (65%)	189 (29%)	< 0.01
Q2. Have you felt unsatisfied because you wanted to play more? (yes)	399 (53%)	65 (70%)	334 (51%)	< 0.01
Q3. Have you been feeling miserable when you were unable to play a game? (yes)	200 (27%)	44 (47%)	156 (24%)	< 0.01
Q4. Were you unable to reduce your time playing games, after others had repeatedly told you to playless? (yes)	285 (38%)	53 (57%)	232 (36%)	< 0.01
Q5. Have you played games so that you would not have to think about annoying things? (yes)	272 (36%)	63 (68%)	209 (32%)	< 0.01
Q6. Have you had arguments with others about the consequences of your gaming behavior? (yes)	273 (37%)	52 (56%)	221 (34%)	< 0.01
Q7. Have you hidden the time you spend on games from others? (yes)	175 (23%)	22 (24%)	153 (23%)	0.96
Q8. Have you lost interest in hobbies or other activities because gaining is all you wanted to do? (yes)	152 (20%)	36 (39%)	116 (18%)	< 0.01
Q9. Have you experienced serious conflicts with family, friends, or partner because of gaming? (yes)	164 (22%)	35 (38%)	129 (20%)	< 0.01
GAMES test total score	3.0 (± 2.6)	4.8 (± 3.0)	2.8 (± 2.5)	< 0.01
DSRS‐C total score	—	7.3 (± 2.1)	—	—
GHQ30 total score	—	9.1 (± 7.0)	—	—
K6 total score	—	—	2.9 (± 4.1)	—
Weekday gaming hours (weekly total)	620.1 (± 604.7)	1271.0 (± 1107.2)	528.4 (± 421.9)	< 0.01
Holiday gaming hours (weekly total)	424.8 (± 384.3)	726.8 (± 591.4)	382.3 (± 324.1)	< 0.01

Abbreviations: DSRS‐C: Birleson Depression Self‐Rating Scale for Children (max = 18 points); GAMES test, GAMing Engagement Screening test (max = 10 points); GHQ30: General Health Questionnaire 30‐item (max = 30 points); IGDS‐C: Internet Gaming Disorder Scale for Children (max = 9 points); K6: Kessler Psychological Distress Scale 6‐item (max = 24 points).

The reliability of the IGDS‐C is detailed in Table 2. All results indicated high internal consistency, with an overall Cronbach's *α* of 0.87 (*n* = 746), 0.80 for clinical participants (*n* = 93), and 0.88 for nonclinical participants (*n* = 653). The ICC was 0.86 for clinical participants (*n* = 23) and 0.61 for nonclinical participants (*n* = 645).

**TABLE 2:** Reliability and validity verification of IGDS‐C results (*N* = 746).

	All (*n* = 746)	Clinical (*n* = 93)	Nonclinical (*n* = 653)
Reliability			
Cronbach's *α*	0.87	0.80	0.88
ICC	0.62	0.86	0.61
Validity			
CFA			
CFI	0.92	0.82	0.91
TLI	0.90	0.76	0.89
RMSEA	0.10	0.12	0.11
SRMR	0.05	0.07	0.05

Abbreviations: CFA: Confirmatory Factor Analysis; CFI: Comparative Fit Index; ICC: Intraclass Correlation Coefficient; RMSEA: Root Mean Square Error of Approximation; SRMR: Standardized Root Mean Square Residual; TLI: Tucker–Lewis index.

**FIGURE 1:**
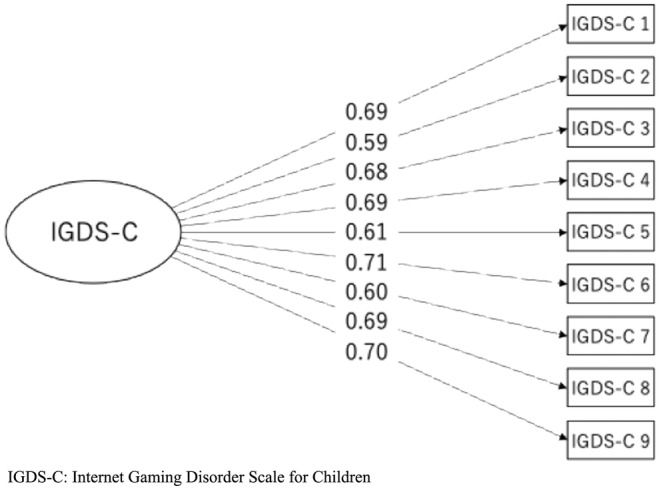
Factor loadings from the IGDS‐C confirmatory factor analysis. (*N* = 746).

The findings investigating validity are outlined in Tables 2 and 3. CFA indicated an overall CFI of 0.92, TLI of 0.90, RMSEA of 0.10, and SRMR of 0.05 (Table 2). Overall factor loadings ranged from 0.59 to 0.71 for all items of the IGDS‐C (Figure 1). The correlation (Spearman's ρ) between the IGDS‐C and GAMES test presented in Table 3 was 0.60 (clinical participants: 0.68, nonclinical participants: 0.57). Correlations between the IGDS‐C and psychological distress scales were also assessed, with results indicating significant values of 0.25 in clinical participants (GHQ30) and 0.35 in nonclinical participants (K6). Gaming hours demonstrated significant correlations with the IGDS‐C on both weekdays (0.34) and holidays (0.39) as well.

**TABLE 3:** Spearman's correlation results of the IGDS‐C total score with other measures (*N* = 746).

	IGDS‐C
GAMES test	
All (*n* = 746)	0.60*
Clinical participants (*n* = 93)	0.68*
Nonclinical participants (*n* = 653)	0.57*
Psychological distress (total score)	
DSRS‐C (clinical participants, 9–12 years old) (*n* = 17)	0.35
GHQ30 (clinical participants, 13–29 years old) (*n* = 76)	0.25*
K6 (nonclinical participants) (*n* = 653)	0.35*
Weekday gaming hours (weekly total)	
All (*n* = 746)	0.34*
Clinical participants (*n* = 93)	0.50*
Nonclinical participants (*n* = 653)	0.27*
Holiday gaming hours (weekly total)	
All (*n* = 746)	0.39*
Clinical participants (*n* = 93)	0.47*
Nonclinical participants (*n* = 653)	0.35*

Abbreviations: DSRS‐C: Birleson Depression Self‐Rating Scale for Children; GAMES test: GAMing Engagement Screening test; GHQ30: General Health Questionnaire 30‐item; K6: Kessler Psychological Distress Scale 6‐item.

* *p* < 0.05.

## 4 Discussion

This study aimed to verify the reliability and validity of the IGDS‐C, a screening instrument for IGD based on the DSM‐5. The IGDS‐C was evaluated in 93 clinical and 653 nonclinical participants to assess its psychometric characteristics across two distinct populations. Participants were largely male, consistent with previous studies suggesting that males are more susceptible to internet gaming and problematic game use [3, 22]. Clinical screening results were consistently greater in the clinical sample, indicating a greater risk of gaming addiction. Both groups generally demonstrated acceptable reliability and validity, although results varied between populations.

Reliability for internal consistency was evaluated using Cronbach's *α*, which measures the interrelatedness and dimensionality of the items within the IGDS‐C. With an acceptable standard *α* value of 0.70 or greater [23], both clinical (*α* = 0.80) and nonclinical (*α* = 0.88) participants showed acceptable internal consistency. For test–retest reliability, both clinical (ICC = 0.86) and nonclinical (ICC = 0.61) participants exhibited moderate to good ICC values representative of reliability [20].

CFA showed mixed validity results, as the GFI met the SRMR criteria but were slightly below CFI, TLI, and RMSEA standards [21]. However, factor loadings for all items of the IGDS‐C were within acceptable ranges (0.59–0.71), displaying moderate to high associations among item scores [21]. Since previous studies have also challenged the dimensionality of the IGDS [7], the IGDS‐C may require further exploration regarding its factorial structure in different Japanese populations. Concurrent validity of the IGDS‐C was verified through correlation with the GAMES test, which has previously established reliability and validity in a Japanese population [14]. Both clinical and nonclinical participants displayed moderate to strong correlations with the GAMES test [24]. The IGDS‐C also demonstrated good convergent validity as it showed significant associations with gaming hours, consistent with findings from the original IGDS [6].

The strengths of this study lie in demonstrating the flexibility of the IGDS‐C in diagnosing IGD, as it confirmed reliability and validity across two distinct populations. However, several limitations also exist. First, recruitment methods of clinical participants through their physicians and nonclinical participants via an online research company may have limited user participation and introduced sampling bias. Additionally, as the principal aim of this study was not intended to compare clinical and non‐clinical participants directly, discussions of group differences in outcomes are restricted due to varying survey design, participant criteria, and sample size. Finally, differences in age and sex within the groups were not considered in this study.

## 5 Conclusion

This study provided evidence supporting the reliability and validity of the IGDS‐C among Japanese clinical and nonclinical participants, suggesting that it is an effective assessment tool that generally reflects the severity of IGD. Future research should further stratify the robustness of the IGDS‐C by investigating its factorial structures, conducting sensitivity and specificity assessments, and examining factors contributing to symptoms of Internet gaming disorder.

## Author Contributions

K.K., T.H., A.T., K.O., M.To., and N.H. designed and conducted the study. M.I. and A.T. supervised the overall execution. K.O. performed the statistical analysis. K.O. and A.T. drafted the manuscript. All authors contributed to interpreting the findings and provided critical review before approving the final manuscript.

## Ethics Statement

Approval of the Research Protocol by an Institutional Reviewer Board: This study was approved by the IRB of both the Tokyo Medical and Dental University (M2022‐322) and the National Center of Neurology and Psychiatry (B2022‐101). Registry and the Registration No. of the Study/Trial: N/A. Animal Studies: N/A.

## Consent

All participants provided informed consent or assent before the survey.

## Conflicts of Interest

The authors declare no conflicts of interest.

## Data Availability

The data sets from this study are unavailable for release as we have not acquired approval from the ethics committee at each institution for data sharing.
